# Paranasal sinus mucoceles and its distortion of craniofacial-orbital anatomy: a narrative synthesis

**DOI:** 10.1007/s00405-024-09174-y

**Published:** 2025-01-17

**Authors:** Okikioluwa Stephen Aladeyelu, Peter Michael Burge, Peterson Makinde Atiba, Anil Madaree, Lelika Lazarus

**Affiliations:** 1 Department of Clinical Anatomy, School of Laboratory Medicine and Medical Sciences, University of KwaZulu- Natal, Durban, South Africa; 2 Department of Plastic and Reconstructive Surgery, School of Clinical Medicine, University of KwaZulu-Natal, Durban, South Africa; 3 Department of Plastic and Reconstructive Surgery, Inkosi Albert Luthuli Central Hospital, Durban, South Africa

**Keywords:** Mucoceles, PNS, Craniofacial, Orbital, Anatomy

## Abstract

**Purpose:**

To explore available literature on PNS mucoceles and its distortions of craniofacial-orbital anatomy with regard to orbital bony defects and ophthalmic manifestations, highlighting the PNS mucoceles that mostly result in these distortions.

**Methods:**

A comprehensive literature search was conducted in June 2024 for available literature on the subject matter viz.; Google Scholar, PubMed and Medline, and Cochrane Library. Eligible studies were subjected to bias assessment. Extracted data from eligible studies were synthesized using narrative synthesis.

**Results:**

Eighteen studies met the inclusion criteria. The 18 studies correspond to a total of 638 patients diagnosed with different types of PNS mucoceles, with males being the highest incidence of patients. Frontal, ethmoid, and fronto-ethmoidal mucoceles had the highest incidences (26.36%, 24.03%, and 23.29%, respectively). Orbital-bony defects, such as disruption and breach of the lamina papyracea, orbital wall erosion, and skull base erosion, were reported as the damage mostly caused by frontal and ethmoidal mucoceles. Ophthalmic manifestations such as proptosis, diplopia, ptosis, preorbital swelling, epiphora, vision problems, and exophthalmos were reported to occur in any type of the PNS mucoceles but are more common in mucoceles in the anterior sinuses.

**Conclusions:**

The narrative review summarizes the distortion of PNS mucoceles to craniofacial-orbital anatomy. Findings from this review will help to create more awareness of the extent of possible distortion that PNS mucoceles could have on craniofacial-orbital anatomy.

## Introduction

Mucoceles, expansive pseudo-cystic tumors of the paranasal sinuses (PNSs), are benign, slow-growing lesions caused by complete ostial obstruction and accumulation of mucous secretions [1–5]. There are various etiological reports on mucoceles. These include obstructive causes (e.g., polyps, trauma, surgery, and tumor compression), inflammatory causes (e.g., infection such as chronic sinusitis, cystic degeneration, and increased mucus secretion), and allergic causes and radiation [6–8]. From a histopathological view, mucoceles appear as mucus-containing cystic lesions covered by pseudostratified columnar respiratory epithelial tissue, which may contain chronic inflammatory cell infiltrates [8].

Despite the benign histological nature of paranasal sinus (PNS) mucoceles, its increasing incidences and aggressiveness toward neighboring structures, such as the orbit, brain, and skull base, have predisposed patients to significant morbidity [3, 9–11]. Often seen in adults (especially in the third and fourth decades of life) and rare in the pediatric population, PNS mucoceles most commonly occur in the frontal, fronto-ethmoidal, and ethmoidal sinuses, accounting for 70–90% of all PNS mucoceles [3, 10, 12].

The PNSs (frontal, ethmoid, maxillary, and sphenoid sinuses), which can also be classified into anterior sinuses (frontal, anterior ethmoid and maxillary) and posterior sinuses (posterior ethmoid and sphenoid), are complex anatomical structures related to their respective bones that make up the craniofacial-orbital skeleton [13–16]. Anatomically, the frontal bone is part of the orbital roof, the maxillary sinus shares the orbit floor, the ethmoid extends along most of the medial wall of the orbit, and the sphenoid almost surrounds the orbital apex [17]. Considering these anatomical relationships, frontal mucocele can extend into the anterior orbit, presenting as a mass, maxillary mucocele may elevate the orbital floor, resulting in proptosis, ethmoidal mucoceles (especially posterior) may cause compression of the orbital apex, sphenoidal mucoceles may extend posteriorly to impinge on the pituitary gland and brainstem [18, 19].

Furthermore, mucoceles can contain purulent secretions, termed chronic pyocoele, and can occasionally be filled with pus, which can rupture, leading to associated osteomyelitis and spread of infection to surrounding anatomical structures [8]. During the pre-antibiotic era, 17% of patients suffering from orbital complications due to rhinosinusitis died of meningitis, and 20.5% became permanently blind in the affected eye [4]. In the same fate, an infected mucocele can induce orbital infection, including orbital subperiosteal abscess, causing facial deformities and may lead to blindness and life-threatening intracranial complications [2–4, 10, 12, 20]. As a result, the treatment and management of patients with PNS mucoceles have evolved around otorhinolaryngologists, with referrals to ophthalmologists and plastic surgeons [14].

Since the first description of mucoceles in the early 1700s [21, 22], various studies (including case reports) have reported mucoceles to occur in all the paranasal sinuses [4, 8, 10–12, 23–28]. However, reports on the defect and distortion of PNS mucoceles of craniofacial-orbital anatomy were limited in these studies. Therefore, this review is undertaken to explore available studies on the craniofacial-orbital distortions of the PNS mucoceles regarding orbital bony defects and ophthalmic manifestations, highlighting the PNS mucoceles that are responsible for these distortions.

## Methods

### Search strategy

A comprehensive literature search was conducted in June 2024 to identify studies that reported craniofacial distortions, orbital defects/complications, and ophthalmic manifestations in relation to PNS mucoceles. Studies were identified by comprehensively searching Google Scholar, PubMed and Medline, and Cochrane Library electronic databases (Access provided by the University of KwaZulu-Natal). The keywords used for this search include paranasal sinus, mucoceles, orbital defects, orbital complications, facial deformities, and ophthalmic manifestations. The Boolean operators “OR”, “AND”, and “NOT” were used to include, restrict, and eliminate search keywords, respectively. A further search was conducted by scanning the reference lists of all relevant articles *(See Appendix)*. It is worth noting that, due to the scarcity of studies addressing the aim of this review and to maximize our chances of retrieving all relevant articles, limitations on the year of publication were not imposed.

### Inclusion criteria

The following inclusion criteria were implemented: (1) articles reporting PNS mucoceles and any related craniofacial-orbital distortion, regardless of the etiology of the PNS mucoceles; (2) articles reporting participants (patients) of any age group, either children or adults (i.e., no restriction to age); (3) articles written in English, already translated into the English language and articles that were available in dual languages, clearly addressing the aim of this review.

### Exclusion criteria

Case studies on PNS mucoceles, reviews, letters to the editors, conference abstracts, or studies containing incomplete or irrelevant data were excluded.

### Study selection

Using a set of questions in line with the study’s objective, two authors (OA and PB) independently screened titles and abstracts of all retrieved studies to assess for eligibility. When eligibility could not be determined from the title or abstract, full articles were retrieved. Also, for studies where full articles were not available for retrieval online, the authors of such articles were directly contacted via email. Due to the limited number of studies identified in relation to the subject matter, the authors decided to include both qualitative and quantitative studies that address the aim of the review. Hence, narrative synthesis was used to synthesize the findings of individual studies as this type of descriptive blending can synthesize findings from both qualitative and quantitative (in this study: morphology and morphometry) studies [29, 30].

### Citation management

All citations of searched articles were imported into EndNote 20.6 (Bld 17174), and duplicate citations were removed manually, with further duplicates removed when found later during the study selection process.

### Risk of bias assessment

The risk of bias (ROB) assessment was performed independently by two assessors (PA & OA) using the Anatomical Quality Assessment (AQUA) Tool (31). The AQUA tool assessed the quality of anatomical studies included in this review using five key domains, viz.: (i) objective(s) and subject characteristics; (ii) study design; (iii) characterization of methods; (iv) descriptive anatomy; and (v) results reporting. Each domain uses some questions to answer Yes, No, or Unclear and ascertain the ROB. Each domain was classified as low risk, high risk, or unclear. The assessors reached a consensus whenever there were disagreements in the scoring of each domain [31].

### Data extraction and analysis

Three authors (OA, PB & PA) developed a data extraction form to extract key information (such as names of the first author and year of publication, study sample, modality of the study, sample size, and key information related to incidences of PNS mucocele and its craniofacial-orbital distortion) from all eligible studies. AM and LL reviewed the data extraction form before being used for data extraction. OA and PB independently used this form to extract data from all the included studies.

Considering the heterogeneity of the included studies in terms of methods, sampling, design, and measures of data presentations and analyses, a narrative synthesis was adopted to synthesize and summarize extracted data from the included studies [29, 30]. In addition, content analysis was done for each paper included in this review by importing data into Microsoft Excel 2016 and using a simple bar chart to present information such as incidences of types of PNS mucoceles identified in the included studies.

## Results

### Description of included study

**Fig. 1:**
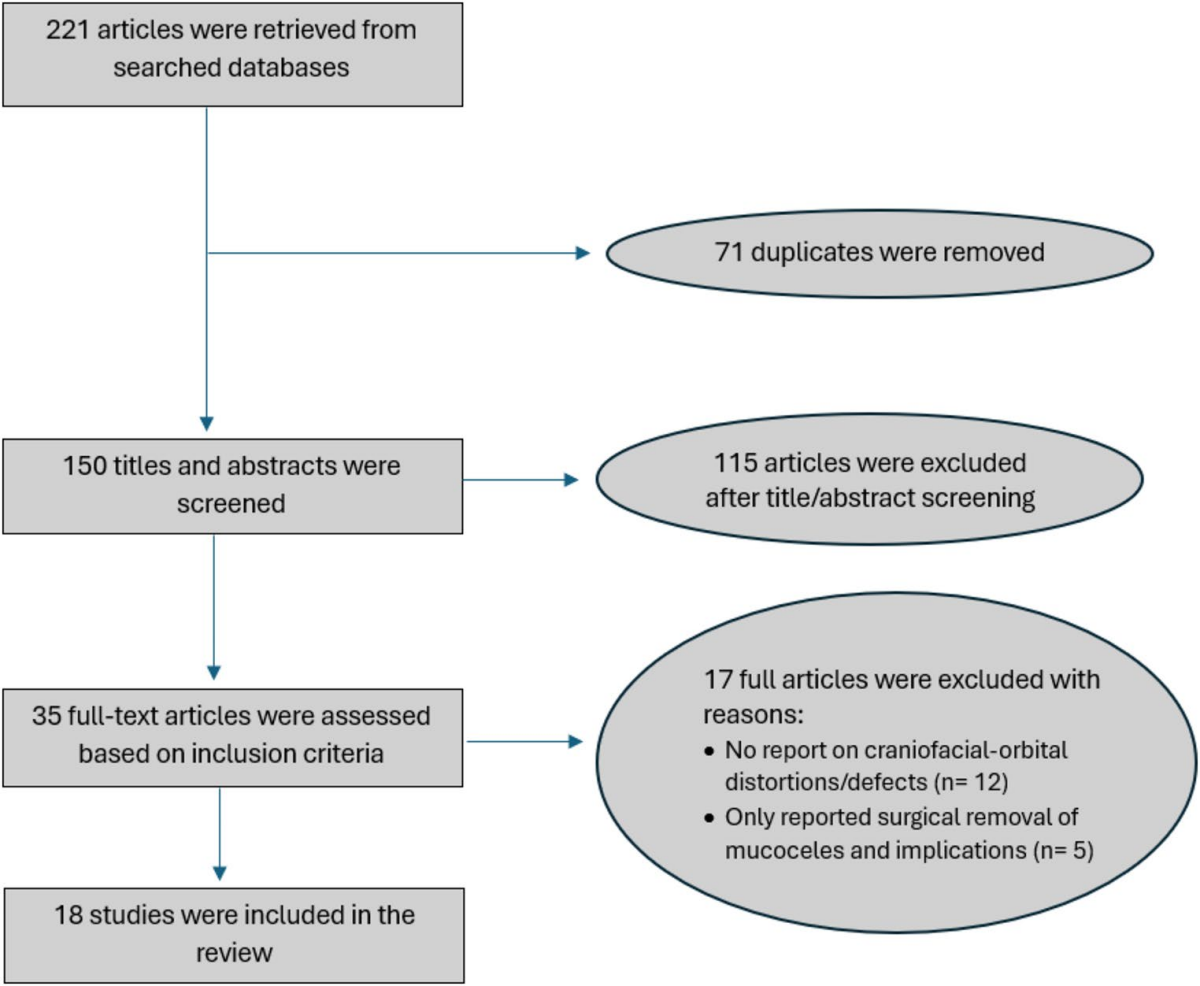
Flow diagram for study selection

A total of 221 articles were identified during the literature search. Seventy-one duplicates were identified and removed, and 150 titles and abstracts were screened. Finally, 35 articles were reviewed, of which 18 met the inclusion criteria of this review (Fig. 1).

Table 1 summarises the included studies and their major findings with regard to craniofacial-orbital distortions and ophthalmic manifestations. All studies were retrospective, reviewing patients’ CT scans, MRIs, and medical records. Three studies each were conducted in the United States of America [32–34] and the United Kingdom [20, 35, 36], two studies each were conducted in India [12, 27] and Taiwan [14, 37], and one study each were from South Korea [38], China [39], Iran [40], Saudi Arabia [41], Japan [4], Italy [42], Tunisia [11], and Morocco [3].

As outlined in Table 1, most of the studies were of morphological assessment of orbital distortion and ophthalmic manifestations, except for the study of Tailor et al. [20] which combined morphological and morphometry assessments. The sex distribution of participants diagnosed with PNS mucoceles in the included studies was given for almost all the studies except for the studies of Makihara et al. [4], Bouatay et al. [11], Van Tassel et al. [32], and Sadiq et al. [35] that did not give information on the sex distributions in their studies. Ten of the included studies had more males in their sex distribution of patients with PNS mucoceles [20, 33, 34, 36–42], three studies had more females [12, 14, 27], while one study gave an equal number of the sex distribution of patients with PNS mucoceles [3].

**Table 1:** Table of characteristics of included studies

Author (Year)	Country (Location)	Sample Size: no. of patients	Male	Female	Age (mean)	Aim of Study	Methodology	Paranasal sinus mucocele(s) identified	Major findings on Craniofacial-orbital distortion/defects/ophthalmic manifestations
Van Tassel et al. (1989)	USA (Houston, Texas)	6 (14 mucoceles)	-	-	-	To present the findings of preoperative MR imaging performed in patients with confirmed mucoceles	MR imaging review of patients with confirmed mucoceles	• Four frontal sinus mucoceles • Four ethmoid sinus mucoceles • Four sphenoid sinus mucoceles • Two maxillary sinus mucoceles	• Bony expansion of mucocele into adjacent orbit in 4 patients (66.7%) with anterior ethmoid and/or frontal sinus involvement.
Har-El (2001)	USA (New York)	103 (108 mucoceles)	52.43%	47.57%	5–83 years	To establish the efficacy of endoscopic management of sinus mucoceles	Retrospective review of 103 patients with 108 mucoceles treated endoscopically between 1988 and 2000	• 66 frontal and fronto-ethmoid sinus mucoceles • 17 ethmoid sinus mucoceles • 7 spheno-ethmoid sinus mucoceles • 12 sphenoid sinus mucoceles • 6 maxillary sinus mucoceles	• Ninety patients (83.3%) intraorbital extension - Proptosis or eye displacement (85 patients; 78.7%) • Skull base erosion with intracranial extension (60 patients; 55.5%)
Tseng et al. (2005)	Taiwan (Taipei)	41	41.5%	58.5%	13–76 years (53.7 years)	To examine the ophthalmic manifestations in patients with paranasal sinus mucoceles, and to evaluate prognosis in terms of the paranasal sinuses involved.	Retrospective review of CT scans and medical charts of patients with paranasal sinus mucoceles and ophthalmic symptoms presentation.	• 25 cases of anterior sinuses (maxillary, frontal, and ethmoidal sinuses) • 11 cases of posterior sinuses (posterior ethmoidal and sphenoid sinuses) • 5 cases of both anterior and posterior sinuses	• Ophthalmic manifestations include proptosis, impairment of ocular mobility, blurred vision, ptosis, and periorbital pain (mostly common in the anterior sinuses than in the posterior sinuses).
Wang et al. (2005)	Taiwan (Taipei)	15	60%	40%	22–76 years (45.5 years)	To report the clinical features of orbital mucoceles and discuss the role of ophthalmologists in the management of patients with orbital mucoceles	Retrospective chart review of all patients with orbital mucoceles	• Frontoethmoidal sinus (6 patients, 40.0%) • Ethmoidal sinus (3 patients, 20.0%) • Frontal sinus (3 patients, 20.0%) • Maxillary sinus (1 patient, 6.7%) • Sphenoidal sinus (2 patients, 13.3%)	• Proptosis (10 patients, 66.7%) • Diplopia (5 patients, 33.3%) • Ocular movement limitation (4 patients, 26.7%) • Ptosis (3 patients, 20.0%) • Decreased visual acuity (3 patients, 20.0%)
Herndon et al. (2007)	USA (Augusta, Georgia)	13	84.62%	15.38%	24–76 years (49.9 years)	To report the presentation and management of extensive fronto-orbito-ethmoid (FOE) mucoceles	Retrospective chart review of patients requiring surgical intervention for FOE mucoceles	• Fronto-orbito-ethmoid sinus mucoceles	• CT demonstrated orbital wall erosion (92.3%) • Destruction and remodeling of the forehead requiring reconstruction (38.5%) • Proptosis (61.5%) • Forehead swelling (38.5%)
Jaswal et al. (2008)	India (Kolkata)	16	43.75%	56.25%	21–75 years (43.68 years)	To evaluate the presenting signs and symptoms, radiological and endoscopic findings, surgical management, and outcomes of patients with paranasal sinus mucoceles	A retrospective study on the paranasal sinus mucoceles managed surgically over a period of 5 years (Mar 2001 to Mar 2006)	• Frontal sinus mucocele (9 cases, 56.25%) • Fronto-ethmoid sinus mucocele (5 cases, 31.25%) • Maxillary sinus mucocele (2 cases, 12.5%)	• Facial deformity (81.25%) • Telecanthus (37.5%) • Proptosis (31.5%) • Erythaema and chemosis of the eye (18.75%) • Discharging sinus in the frontal region (6.25%)
Mohammadi et al. (2008)	Iran (Tabriz)	18	55.6%	44.4%	29–72 years	To review endoscopic surgical treatment of paranasal sinus mucocele	Retrospective review patients’ preoperative coronal and axial CT scans.	• Fronto-ethmoidal complex (33%) • Ethmoid sinus (22%) • Maxillary sinus (11%) • Sphenoid sinus (11%)	• 33% of patients were reported to have diplopia. • 22% of patients had orbital displacement and visual deficiency
Sadiq et al. (2009)	United Kingdom (Manchester)	45	-	-	-	To investigate the ophthalmic manifestations of paranasal sinus mucoceles	A retrospective chart review of all patients diagnosed with paranasal sinus mucoceles	• Frontal (35 patients, 70%) • Ethmoid (6 patients, 12%) • Fronto-ethmoid (2 patients, 4%) • Maxillary (3 patients, 6%) • Sphenoid (4 patients, 8%)	• Periorbital swelling (29 patients, 64.4%) • Diplopia (9 patients, 20%) • Proptosis (6 patients, 13.3%) • Epiphora (1 patient, 2.2%) • No ophthalmic manifestations (3 patients, 6.7%)
Hssaine et al. (2016)	Morocco (Marrakech)	32	50%	50%	(43.28 years)	To study the epidemiological, clinical, therapeutic, and evolution aspects of this pathology	A retrospective study of patients’ CTs and MRIs operated on and followed for mucocele	• Fronto-ethmoidal sinus (27 cases) • Maxillary sinus (3 cases) • Sphenoid sinus (2 cases)	• Most common orbital defects were periorbital swelling and exophthalmia with the highest frequency occurring in fronto-ethmoidal sinus mucocele
Tailor et al. (2016)	United Kingdom (Birmingham)	15	80%	20%	32–80 years (55 years)	To describe the clinical features of orbital involvement arising from occult obstructive frontal sinus disease and to highlight key features to aid diagnosis.	A retrospective review of case notes and CT imaging performed for consecutive patients diagnosed with fronto-orbital mucocele (FOM).	• Fronto-ethmoidal sinus (100%)	• Majority (14 out of 15 patients) presented with upper lid erythema and swelling. • Exophthalmometry was documented for 11 patients, all of whom were noted to have proptosis ranging from 2 to 7 mm. • Homogeneous, well-defined expansions of the sinus into the superior orbit.
Bouatay et al. (2019)	Tunisia (Monastir)	16			15–83 years (47 years)	To study the radiological characteristics of mucocele on CT scan and MRI	A retrospective study of patients with mucoceles explored by imaging and operated on by the author’s department	• 13 cases (81%) present at the fronto-ethmoidal complex • 2 cases (13%) in the maxillary sinus • 1 case (6%) in the sphenoidal sinus	• Bone lysis in all patients • Distortion of lamina papyracea in 6 cases (38%) • Intra-orbital extension in 9 cases (7 fronto-ethmoidal, 1 sphenoidal, 1 maxillary mucocele)
Makihara et al. (2020)	Japan (Kagawa)	82 (92 sides)			28–29 years (64.6 years)	To present experiences in endoscopic surgical management of orbital complications secondary to infected paranasal sinus mucoceles.	Retrospective review of the medical charts, CTs, and MRIs of 82 patients who had 92 sides diagnosed with paranasal sinus mucoceles	• Ethmoid sinus = 9/92 (9.8%) • Frontal sinus = 7/92 (7.6%) • Maxillary sinus = 62/92 (67.4%) Sphenoid sinus = 2/92 (2.2%) • Maxillary-ethmoid = 3/92 (3/3%) • Ethmoid-frontal = 9/92 (9.8%)	• 7/92 sides diagnosed had distortion/defects of craniofacial-orbital anatomy in mucoceles involving the maxillary sinus and ethmoid-frontal complex only. (3 males; 4 females) (64-91 ears; µ = 75.8 years) • Of the maxillary sinus mucocele, 1/62 had partial defect of orbital wall bone with subperiosteal edema- Type II orbital complication of sinusitis. • Of mucoceles involving the ethmoid-frontal complex, • 6/62 had partial defect of orbital wall bone with preseptal cellulitis (1/6)- Type I, Subperiosteal edema (2/6)- Type II, Subperiosteal abscess (3/6)- Type III of orbital complications of sinusitis
Kim et al. (2021)	South Korea (Busan)	52	71.15%	28.85%	37–69 years	To investigate the clinical and radiological features affecting the ophthalmic manifestations in patients with paranasal sinus mucoceles involving the orbit	A retrospective review of clinical parameters, medical charts, and CTs of 52 patients who underwent endoscopic sinus surgery of paranasal sinus mucoceles.	• Anterior ethmoid sinus (48.1%) • Posterior ethmoid sinus (17.3%) • Maxillary sines (38.5%) • Frontal sinus (36.5%) Sphenoid sinus (9.6%)	Ophthalmic manifestations were significantly high in mucoceles involving frontal & ethmoidal sinuses but significantly lower in maxillary sinus involvement. These include: -Exophthalmos (38.5%) -Visual disturbance (25%) -Ocular pain (25%) -Diplopia (23.1%)
Rampinelli et al. (2021)	Italy (Brescia)	34 (after a review of 140 patients)	70.6%	29.4%	1.2–8 years (7.7 years)	To identify PNS mucoceles in the personal large series children with cystic fibrosis and to assess their diagnosis and treatment.	A retrospective review of medical records and CT scans of children diagnosed with cystic fibrosis and paranasal mucocele	• 29 cases of maxillary sinus (17 bilateral; 12 unilateral) • 4 cases of ethmoidal sinus (unilateral only) • 1 case of sphenoid sinus (unilateral only)	• Ophthalmic manifestation includes retro-orbital pain and/or diplopia (8.8%)
Shanbag et al. (2022)	Indian (Dharwad, Karnataka)	21	42.86%	57.14%	9–69 years (42.95 years)	To describe the clinical profile, intraoperative experience and outcomes of a series of patients who underwent surgical treatment for paranasal sinus mucoceles.	Retrospective review of patients’ CTs and MRIs	• Frontal (42.85%) • Fronto-ethmoidal sinus (38.09%) • Ethmoid sinus (14.28%) • Sphenoid sinus (4.76%)	• 9/21 patients had orbital extension: -Superior orbital erosion of frontal mucocele (5/9) -breach of lamina papyracea (4/9) • 3/21 patients had skull base erosion: -erosion of the ethmoid/fovea (2/3) -posterior extension eroding into frontal lobe (1/2) • 3/21 patients had visual symptoms (diplopia): -Excessive watering (2/3) -blurring of vision (1/3)
Magboul et al. (2023)	Saudi Arabia (Abha)	8	75%	25%	14–67 years	To identify clinical presentation, most common paranasal sinus affected by mucocele, management, and the rate of recurrence.	A retrospective, hospital file-based study and review of CTs/MRIs of 8 reoccurring cases of paranasal sinus mucoceles.	• Frontal sinus mucocele (40%; either left or right) • Ethmoid sinus (25%; either left or right)	Craniofacial-orbital distortion/defects include: -Orbital swelling (5/8) -Epiphora (3/8) -Ptosis (1/8) -Double vision (4/8)
Malik et al. (2023)	United Kingdom (London)	60 (after a review of 171 patients)	63%	37%	3–89 years (51 years)	To evaluate ophthalmic features and outcomes for patients with sinonasal mucoceles expanding into the orbit.	A retrospective review of patients’ CT images and diagnostic database.	• 58 unilateral and 2 bilateral paranasal sinus mucoceles were reported. • 97% were of frontal and/or ethmoidal sinus origin	• Bony expansion of the paranasal sinus was common (98%), associated with bone erosion (96%) • Disruption of lamina papyracea, associated with diplopia and proptosis (69%) • Orbital fat inflammation and enlargement of the adjacent extraocular muscles (17% & 6%, respectively)
Peng et al. (2023)	China (Tianjin)	61	55.7%	44.3%	10–77 years	To describe the ocular symptoms in a series of patients with nasal sinus mucoceles of different locations.	Retrospective review CT images of patient diagnosed (treated) with sinus mucoceles and ocular symptoms	• Frontal sinus (8/61) • Ethmoid sinus (25/61) • Sphenoid sinus (28/61)	Most ophthalmic manifestations include exophthalmos or displacement, eye pain, blindness or decreased vision, blepharoptosis, and diplopia

### Risk of bias assessment of the included studies

The ROB assessment for each study is summarized in Table 2. Domain 1 (objective(s) and subject characteristics), all (100%) of the included studies were of low ROB [3, 4, 11, 12, 14, 20, 27, 32–42]. Domain 2 (study design), all (100%) of the included studies were of low ROB [3, 4, 11, 12, 14, 20, 27, 32–37, 39–43]. Domain 3 (characterization of methods), all (100%) of the included studies were of high ROB [3, 4, 11, 12, 14, 20, 27, 32–42]. Domain 4 (descriptive anatomy), 94.44% of the included studies had low ROB [3, 4, 11, 12, 14, 20, 27, 32, 33, 35–42, and 5.56% had high ROB [34]. Domain 5 (results reporting), 94.44% of the included studies had a low ROB [3, 4, 11, 12, 14, 20, 27, 32, 33, 35–42], and 5.56% had a high ROB [34].

**Table 2:** Risk of bias assessment

	Author (Year)	Domain 1 Objective(s) and subject characteristics	Domain 2 Study design	Domain 3 Characterization of methods	Domain 4 Descriptive anatomy	Domain 5 Results reporting
1	Van Tassel et al. (1989)	Low	Low	High	Low	Low
2	Har-El (2001)	Low	Low	High	Low	Low
3	Tseng et al. (2005)	Low	Low	High	Low	Low
4	Wang et al. (2005)	Low	Low	High	Low	Low
5	Herndon et al. (2007)	Low	Low	High	High	High
6	Jaswal et al. (2008)	Low	Low	High	Low	Low
7	Mohammadi et al. (2008)	Low	Low	High	Low	Low
8	Sadiq et al. (2009)	Low	Low	High	Low	Low
9	Hssaine et al. (2016)	Low	Low	High	Low	Low
10	Tailor et al. (2016)	Low	Low	High	Low	Low
11	Bouatay et al. (2019)	Low	Low	High	Low	Low
12	Makihara et al. (2020)	Low	Low	High	Low	Low
13	Kim et al. (2021)	Low	Low	High	Low	Low
14	Rampinelli et al. (2021)	Low	Low	High	Low	Low
15	Shanbag et al. (2022)	Low	Low	High	Low	Low
16	Magboul et al. (2023)	Low	Low	High	Low	Low
17	Malik et al. (2023)	Low	Low	High	Low	Low
18	Peng et al. (2023)	Low	Low	High	Low	Low

### PNS mucoceles identified in the included studies

**Fig. 2:**
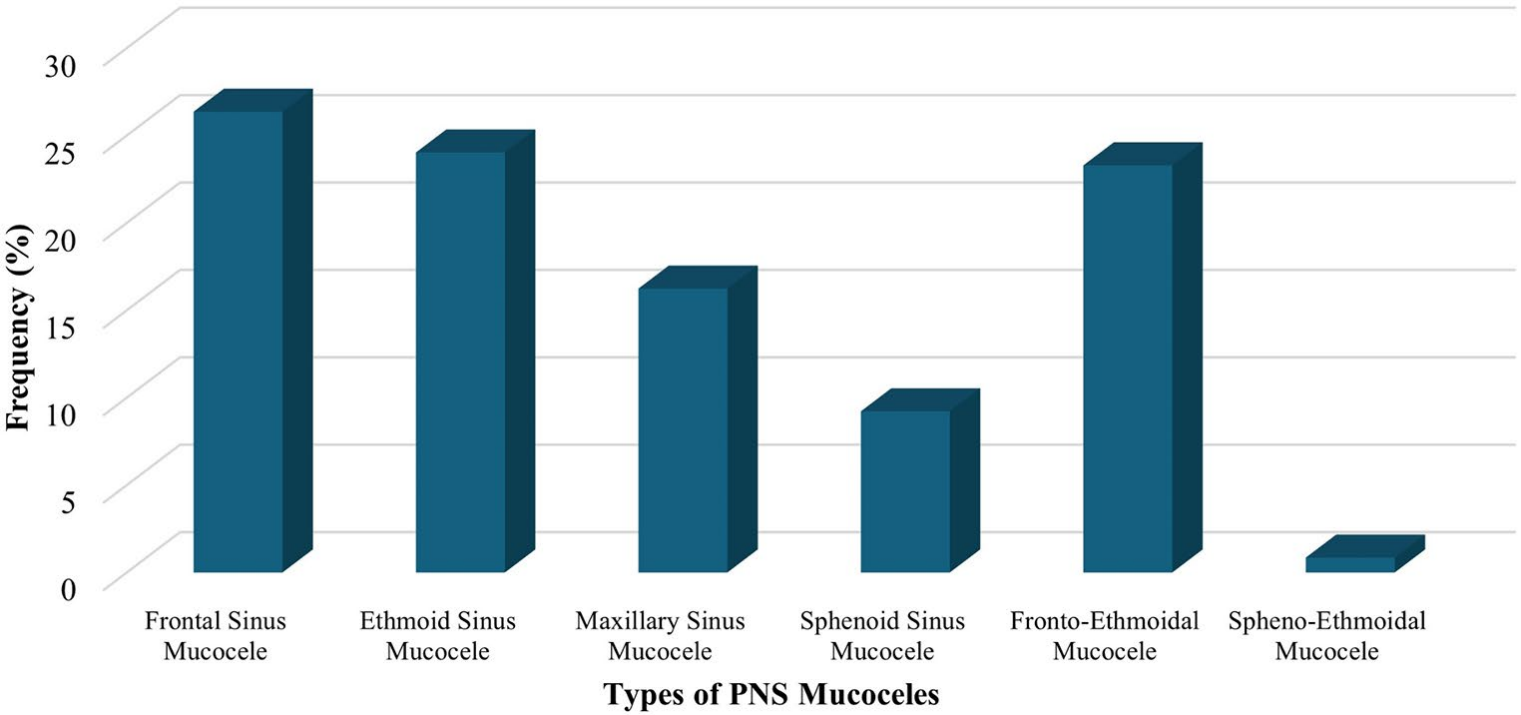
Bar chart showing incidences of the types of PNS mucoceles identified in the included studies

From the 18 studies included in this review, a total of 638 patients (661 sides) were identified to be diagnosed with different types of PNS mucoceles, with males being the highest incidence of patients. Frontal sinus, ethmoid sinus, and fronto-ethmoidal mucoceles had the highest incidences (26.36%, 24.03%, and 23.29%, respectively), while the spheno-ethmoidal mucoceles had the lowest incidence (Fig. 2).

### PNS mucoceles and orbital bony defect

Craniofacial-orbital bony defects were reported in seven studies, mostly occurring in patients with frontal and ethmoidal sinus mucoceles [4, 11, 12, 32–34, 36]. Distortion, disruption, and breach of the lamina papyracea (the thin bony plate that forms the medial wall of the orbit), orbital wall erosion, and skull base erosion were the most reported craniofacial-orbital bony defects caused by PNS mucoceles [11, 12, 33, 36]. Intra-orbital extension, orbital bone expansion, and destruction and remodelling of the forehead requiring reconstruction were also reported [32–34]. Furthermore, an incidence of 97% of orbital involvement with intracranial extension and skull-base erosion was documented [12, 33, 34] (Table 1).

### PNS mucoceles and ophthalmic manifestations

Different types of ophthalmic manifestations and complications were reported in all the 18 studies included in this review, having to occur in any type of the PNS mucoceles (but more common in mucoceles in the anterior sinuses). Almost all the included studies reported proptosis, diplopia, ptosis, preorbital swelling, and exophthalmos. Few studies reported vision problems/symptoms, ocular movement problems, and epiphora [12, 14, 37, 39, 41], while Makihara et al. [4] reported Type I, II & III orbital complications of sinusitis.

## Discussion

The aim of this narrative review was to explore the craniofacial-orbital anatomical distortion caused by PNS mucoceles. Several factors can predispose one to PNS mucoceles. These include previous paranasal sinus surgery, history of facial trauma, fibrosis, inflammation, paranasal sinus polyposis, or a mass effect caused by malignancy, osteoma, fibrous dysplasia, Paget’s disease [8]. Although been reported as a benign and expansive pseudo cystic slow-growing paranasal sinus formation, mucoceles can have many negative consequences, such as distorting local anatomy and exerting pressure on adjacent structures as they enlarge [8, 11]. As a result of the very close anatomic proximities of PNSs to the orbit and their anatomical bony-relations, the pathologic process of the PNS mucoceles could easily affect the orbital bones and the eyes, resulting in various clinical manifestations such as headache, maxillofacial pressure, nasal congestion, nasal obstruction, proptosis, globe displacement, diplopia, eyelid swelling, pain, restricted extraocular motility, and optic neuropathy [19].

In the current review, findings concerning the distortion of craniofacial-orbital anatomy and ophthalmic manifestations resulting from PNS mucoceles were revealed. Although Scangas et al. [22], identified frontal and frontoethmoidal mucoceles as the most common sinuses reported, with the ethmoid, maxillary, and sphenoid sinuses less frequently involved, the present review identified the frontal sinus, ethmoid, and fronto-ethmoidal mucoceles as the most commonly reported with maxillary sinus mucoceles having higher incidences than the sphenoid sinus and spheno-ethmoidal mucoceles. Although Dikici et al. [8] stated no sex preference for mucoceles, Hssaine et al. [3] stated a slight male predominance, while Bouatay et al. [11] identified a sex ratio of 1.14 in their case series. The present review showcased a male predominance of PNS mucoceles, hence supporting the claims of Hssaine et al. [3] and Bouatay et al. [11].

PNS mucoceles present with different kinds of symptoms depending on the sinus involved, the location of the mucocele, and its volume; however, the onset of the clinical symptoms of mucocele is usually delayed [3]. This delay, however, is one major factor contributing to the late presentation and diagnosis of mucoceles in patients, resulting in the damage, obstruction, distortion, and defect of the anatomy of neighboring structures (except for those that reported to the hospital for other related cases and the benign mass was discovered incidentally during routine radiological investigations) [19]. Hence, their early detection is very important [11]. In this review, the disruption and breach of the thin bony plate that forms the medial wall of the orbit, the lamina papyracea, as well as orbital wall erosion, skull base erosion, orbital bone expansion, and destruction and remodelling of forehead that required reconstruction were the result of damage to the related-bones of the orbits caused by mucoceles mostly involving the frontal and fronto-ethmoidal sinuses. Intracranial extension was also reported, with almost all of the cases having orbital involvement.

These bony distortions and disruptions are caused by a combination of both mechanical and biochemical forces [22, 44–47]. The increased pressure within the mucocele cavity causes stress-induced bony remodeling, at this same time, chronic inflammation within the obstructed sinus leads to the release of cytokines, collagenases, and prostaglandins, which stimulate osteoclastic bone resorption [44–47].

Furthermore, different types of eye-related defects were identified in the course of this review, occurring regardless of the sinus involved and the location of the mucocele; however, they are more common in mucoceles involving the anterior sinuses- frontal, anterior ethmoid, and maxillary sinuses. The present review identified proptosis, diplopia, ptosis, preorbital swelling, and exophthalmos as the most common eye-related defects caused by PNS mucoceles. These reveal the extent of damage mucocele exact of local anatomy [2, 4, 14, 19]. Other eye-related defects and ophthalmic manifestations reported in this review include visual disturbances, ocular movement problems, epiphora, and Type I, II, and III orbital complications of sinusitis as classified by Hubert [48] and modified by Chandler et al. [23].

### Strengths and limitations

To the best of our knowledge, this is the first review that systematically summarized studies on PNS mucoceles and its distortion of craniofacial-orbital anatomy, reporting the damages and disruptions to related bones of the orbital anatomy, eye-related defects and ophthalmic manifestations. This review has been strengthened by its extensive database searches and the quality assessment of included studies. However, there are a few limitations: (i) only a few studies met the inclusion criteria, resulting in only 18 studies included in this review; (ii) the exclusion of unpublished reports, review articles, congress presentations, and case studies might have led to having the included studies to be limited to USA, Europe, East-, West- and South- Asia, and North Africa, which might result in the omission of certain information and reports from other geographical regions of the world.

### Conclusion and recommendations

The authors summarize the distortion of PNS mucoceles to craniofacial-orbital anatomy. Firstly, mucoceles were identified to occur in all the PNSs, with frontal sinus, ethmoid, and fronto-ethmoidal sinuses having the highest incidences. Secondly, this narrative review identified different damages caused by PNS mucoceles to the relative bones that make up the anatomy of the orbit to be mostly attributed to frontal and fronto-ethmoidal mucoceles. Lastly, PNS mucoceles were identified to develop at least one eye-related defect, regardless of the sinus involved and the location of the mucocele. Findings from this review will help to create more awareness concerning the extent of possible distortion that PNS mucoceles could have on the craniofacial-orbital anatomy. To the patient, it will help increase knowledge about reporting to the nearest clinic whenever any of the ophthalmic manifestations reported in this review are identified, as PNS mucocele can be an underlying cause. To the clinician, it will raise the index of suspicion for the diagnosis of mucoceles as well as to which PNS could be involved based on the craniofacial-orbital distortion presented to them by their patients.

## Appendix: database search strategy

**Table:**

**Electronic database search using search terms/keywords:**				
**Search Engines/Database**	**Search Date (2024)**	**Search Terms (Keywords used with Boolean operators)**		**Number of Articles Identified**
Google Scholar	June 3rd to June 4th	“Paranasal sinus and mucoceles” “Mucoceles and orbital defects or orbital complications” “Paranasal sinus mucoceles and facial deformities” “Paranasal sinus mucoceles not mucoceles of the lips” “Paranasal sinus mucocele and ophthalmic manifestations		77
PubMed and Medline	June 6th to June 7th	“Paranasal sinus and mucoceles” “Mucoceles and orbital defects or orbital complications” “Paranasal sinus mucoceles and facial deformities” “Paranasal sinus mucoceles not mucoceles of the lips” “Paranasal sinus mucocele and ophthalmic manifestations		47
Cochrane Library	June 10th to June 11th	“Paranasal sinus and mucoceles” “Mucoceles and orbital defects or orbital complications” “Paranasal sinus mucoceles and facial deformities” “Paranasal sinus mucoceles not mucoceles of the lips” “Paranasal sinus mucocele and ophthalmic manifestations		38
**Total**	**162**			
**Electronic database search using reference lists of all relevant articles**				
**Search Engines**	**Search Date (2024)**	**Random Search (using reference lists of all relevant articles)**		**Number of Articles Identified**
Google Scholar	June 19			26
PubMed and Medline	June 21	“Reference lists of all relevant articles”		19
Cochrane Library	June 25	“Reference lists of all relevant articles”		14
**Total**	**59**			

**Table:**

Articles identified using keywords with the use of Boolean operators	162
Article identified using reference lists of all relevant articles	59
**Total Number of Articles Identified**	**221**
